# Supplementary material for: Essential Paralogous Proteins as Potential Antibiotic Multitargets in Escherichia coli
Source: Microbiol Spectr. 2022 Nov 29;10(6):e02043-22. doi: 10.1128/spectrum.02043-22 (PMC9769728; doi:10.1128/spectrum.02043-22)
Supplement: Supplemental 2 — . Download spectrum.02043-22-s0002.pdf, file, 0.8 MB [file spectrum.02043-22-s0002.pdf]

Supplemental Material

Table S1: List of all *E. coli* Sakai protein sequences

Table S2: List of *E. coli* essential proteins used in this study

Table S3: Paralog analysis of *E. coli* Sakai proteins

Table S4: Essential proteins with *E. coli*-conserved paralogs

Table S5: Bacterial conservation analysis

Table S6: Available target protein structures

Text S1: Contains scripts and instructions to run scripts used in this manuscript. Includes the following:

- **README.txt** Instructions to run Python programs used in this manuscript.
- **EcoliProteinsBlast.py** Python program that sequentially BLASTs (tBLASTn) each *E. coli* Sakai protein against the NCBI nr database, restricted to *E. coli* sequences.
- **EcoliParalogs.py** Python program that tabulates output data from EcoliProteinsBlast.py to create a table of the total number of *E. coli* genomes having hsp numbers of 1, 2, 3, 4-9, or >=10 for each protein.

Dataset S1: SakaiProteins.xls Dataset used as input for EcoliProteinsBlast.py.

Text S1

CHardy_EcoliEssParalogs_README.txt

There are two Python programs and two text files in the Supplemental Material for this manuscript:

EcoliProteinsBlast.py

EcoliParalogs.py

CHardy_EcoliEssParalogs_README.txt

SakaiProteins.txt (if in .xlsx format, first export as a tab-delimited .txt file named "SakaiProteins.txt")

The two Python scripts should be saved individually, and the SakaiProteins.txt file should be placed with them in the same folder. Use the following steps to run these programs:

Step 0.

pip install biopython

pip install xmltodict

Step 1.

python3 EcoliProteinsBlast.py

This program sequentially BLASTs (tBLASTn) each protein from "SakaiProteins.txt" against the NCBI nr database restricted to E. coli, and outputs certain data to tab-delimited text files. One file is produced for each protein as numbered in the SakaiProteins.txt file. For example, the first file produced will be: "Sakai_BLAST_Results_1.txt".

If an error occurs, which happens periodically possibly due to errors on the NCBI end, restart the program at the desired record number by changing the integer in "records[0:]:". According to standard Python numbering, 0 corresponds to record 1, 1 corresponds to record 2 etc.

Step 2.

python3 EcoliParalogs.py

This program reads in all the Sakai_BLAST_Results_[id].txt files and produces "SakaiParalogs_Summary.txt", a list of total number of E. coli genomes having hsp numbers of 1, 2, 3, 4-9, or >=10 for each protein. It also produces a column counting the number of hsps in the E. coli Sakai genome for each protein ("Sakai hsp_num). The "Total entries" column counts the total number of matches found for all E. coli genomes.

EcoliProteinsBlast.py

```
#EcoliProteinsBlast.py

#Blasts protein sequences from E. coli Sakai against E. coli genomes and outputs results as tab-delimited txt files


import Bio

import xmltodict

import csv

from Bio import SeqIO

from Bio.Blast import NCBIWWW

from Bio.Blast import NCBIXML


records = list(SeqIO.parse("SakaiProteins.txt", "tab"))


for record in records[0:]:

    print(record.id)

    result_handle = NCBIWWW.qblast("tblastn", "nr", str(record.seq), expect = 0.001, hitlist_size = 20000,
entrez_query = '"Escherichia coli" [Organism]')

    xml_string = result_handle.read()

    result_handle.close()

    record_data = xmltodict.parse(xml_string)

    hits = record_data["BlastOutput"]["BlastOutput_iterations"]["Iteration"]["Iteration_hits"]["Hit"]

    with open ("Sakai_BLAST_Results_" + str(record.id) + ".txt", "w") as tabfile:

        tabwriter = csv.writer(tabfile, delimiter = "\t")

        tabwriter.writerow(["Hit_num", "Hit_id", "Hit_def", "Hit_accession", "Hsp_num", "Hsp_evalue",
"Hsp_identity", "Hsp_positive", "Hsp_align-len"])

        for hit in hits:

            hsps = hit["Hit_hsps"]["Hsp"]

            if not (type(hsps) is list):

                hsps = [hsps]

            for hsp in hsps:

                tabwriter.writerow([hit["Hit_num"], hit["Hit_id"], hit["Hit_def"], hit["Hit_accession"], hsp["Hsp_num"],
hsp["Hsp_evalue"], hsp["Hsp_identity"], hsp["Hsp_positive"], hsp["Hsp_align-len"]])
```

EcoliParalogs.py

```
#EcoliParalogs.py

#Counts the number of E. coli genomes with hsp=1, 2, 3, 4-9, and 10+


import csv

import os


with open ("SakaiParalogs_Summary.txt", "w") as writefile:

    tabwriter = csv.writer(writefile, delimiter = "\t")

    tabwriter.writerow(["Sequence No.", "Total entries", "Sakai hsp_num", "No. hsp = 1", "No. hsp = 2", "No. hsp = 
3", "No. hsp = 4-9", "No. hsp >10"])


    for i in range(1, 5204):

        try:

            with open ("Sakai_BLAST_Results_" + str(i) + ".txt", "r") as readfile:

                reader = csv.reader(readfile, delimiter="\t")

                header = reader.__next__()

                count_total = 0

                count_Sakai = 0

                count_hsp1 = 0

                count_hsp2 = 0

                count_hsp3 = 0

                count_hsp4_9 = 0

                count_hsp10plus = 0


                for row in reader:

                    if len(row) > 0:

                        count_total = count_total + 1

                        if "Escherichia coli O157:H7 str. Sakai DNA, complete genome" in row[2]:

                            count_Sakai = count_Sakai + 1

                        if int(row[4]) == 1:

                            count_hsp1 = count_hsp1+ 1
```

```
                    if int(row[4]) == 2:
                        count_hsp2 = count_hsp2+ 1
                    if int(row[4]) == 3:
                        count_hsp3 = count_hsp3 + 1
                    if int(row[4]) >=4 and int(row[4]) <=9:
                        count_hsp4_9 = count_hsp4_9 + 1
                    if int(row[4]) >= 10:
                        count_hsp10plus = count_hsp10plus + 1

                tabwriter.writerow([str(i), count_total, count_Sakai, count_hsp1, count_hsp2, count_hsp3, count_hsp4_9, count_hsp10plus])

        except FileNotFoundError as err:
            print(i, "File not found")
```
